# Risk-adapted management for vasa praevia: a retrospective study about individualized timing of caesarean section

**DOI:** 10.1007/s00404-019-05125-9

**Published:** 2019-03-26

**Authors:** Gülen Yerlikaya-Schatten, Kinga M. Chalubinski, Sophie Pils, Stephanie Springer, Johannes Ott

**Affiliations:** Division of Obstetrics and Feto-Maternal Medicine, Department of Obstetrics and Gynecology, Medical University of Vienna, Waehringer Guertel 18-20, 1090 Vienna, Austria

**Keywords:** Vasa praevia, Individual management, Preterm delivery, Caesarean section

## Abstract

**Purpose:**

Vasa praevia is a rare condition with high foetal mortality if not detected prenatally. There is limited evidence available to determine the ideal timing of delivery and management recommendations. The aim of this study was to critically review our experience with vasa praevia, with a focus on diagnosis and management.

**Methods:**

In a retrospective analysis, all cases of vasa praevia identified in our department from January 2003 to December 2017 were included. All cases were diagnosed antenatally during sonographic inspection of the placenta, and individualized management for each patient was performed based on individual risk factors. 19 cases of vasa praevia were identified (15 singletons, four twins). 13 patients (79%) presented placental anomalies. In patients at high risk for preterm birth, caesarean delivery was performed between 34–35 weeks after early hospitalization and administration of corticosteroids, whereas in patients at low risk for preterm birth, caesarean section could be delayed to 35–37 weeks of gestation. Administration of corticosteroids was not obligatory in the latter cases.

**Results:**

There were two acute caesarean sections, due to premature abruption of the placenta and vaginal bleeding. There was no maternal or foetal/neonatal death. None of the neonates required blood transfusion. There is limited evidence available with which to determine the ideal timing of delivery.

**Conclusion:**

However, our individualized, risk-adapted management, which attempts to delay the timing of caesarean section up to two weeks beyond the standard recommendation, seems feasible, with just two emergency caesarean sections and no case of foetal or maternal death.

## Introduction

**Fig. 1:**
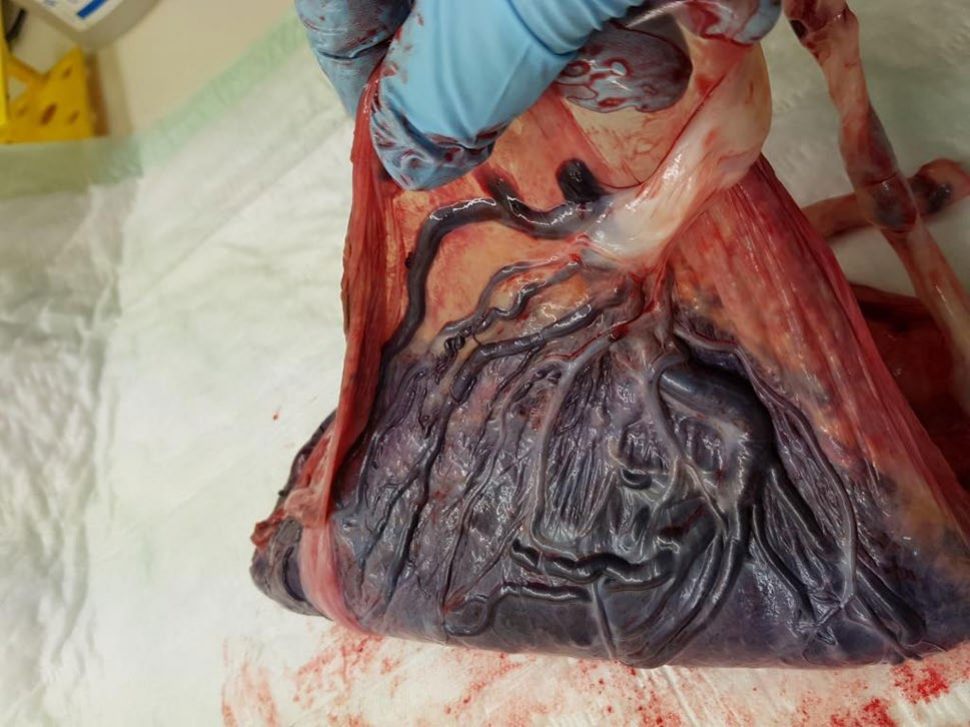
Placenta with a velamentous cord insertion and vasa praevia. The foetal blood vessels in the membranes are the vessels which cover the internal os of the cervix and under the foetal presenting part during pregnancy, without the protection of Wharton’s jelly

Vasa praevia is defined as the presence of foetal blood vessels in the membranes covering the internal os of the cervix and under the foetal presenting part, without the protection of Wharton’s jelly (Fig. 1) [1–3]. Approximately, 1 in 2500 pregnancies is complicated by vasa praevia [4]. It is a condition with a high foetal mortality if rupture of the membranes occurs, since it can lead to foetal death due to exsanguination in 50–100% of cases. Of the surviving newborns, more than 50% require blood transfusion [3]. Potential risk factors for vasa praevia are placenta abnormalities, such as placenta praevia, bilobed or succenturiate, placenta, umbilical cord insertion in the lower third part of the uterus at first-trimester ultrasound and velamentous cord insertion. Furthermore, pregnancies conceived by assisted reproductive technologies are at high risk for vasa praevia [5].

**Fig. 2:**
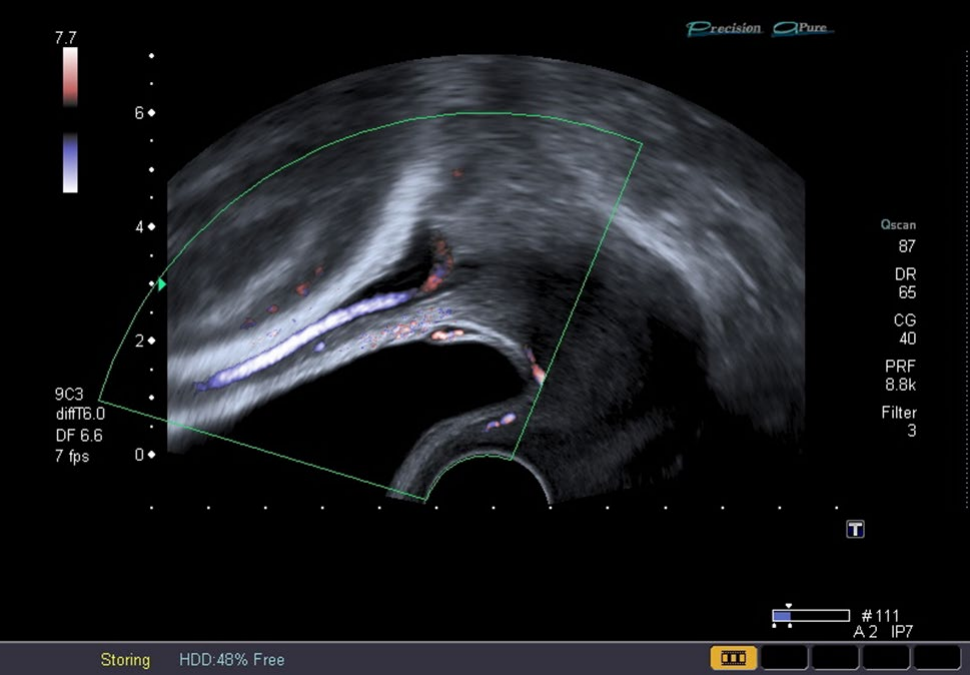
Vasa praevia: transvaginal view and Color Doppler mode. The presence of foetal blood vessels covering the internal os of the cervix and under the foetal presenting part

**Fig. 3:**
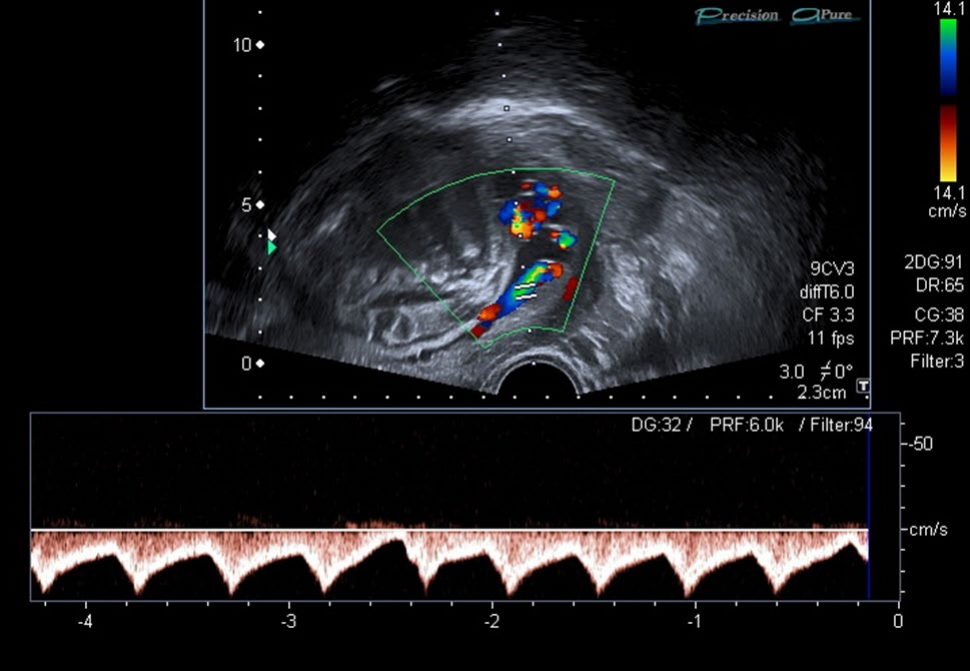
Vasa praevia: Color Doppler measurement. The vessels in front of the internal os of the cervix show typical umbilical waves form as it appears on a free loop of the umbilical cord, which is a proof for vasa praevia

Through prenatal diagnosis of vasa praevia by ultrasound examination (Figs. 2 and 3), neonatal outcome can be improved due to caesarean delivery before rupture of membranes at 35 weeks of gestation, after the administration of corticosteroids for foetal lung maturation, as recommended by previous studies [3, 6, 7]. Due to the fact that vasa praevia can be detected at an early stage of pregnancy, the legitimate question arises whether the pregnancy can be prolonged under close control to prevent iatrogenic prematurity and their consequences. Thus, the objective of this retrospective case series was to critically review the experience with our management approach, which is adapted to the patients’ individual situation and does not include routine early caesarean section.

## Methods

This retrospective case study was performed at the Department of Obstetrics and Fetomaternal Medicine, after approval by the local Institutional Review Board. According to the ethics committee of the Medical University of Vienna, informed consent of the patient is not necessary. Data analysis was performed by retrospective chart review. In our department, the PIA Fetal Database software (GE-Viewpoint, Wessling, Germany) is used as the basic perinatologic database. All cases with vasa praevia from January 2003—to December 2017 were included. The ultrasound machines used for the examinations were Power Vision and Aplio MX (Toshiba, Japan). Examinations were performed transvaginally and transabdominally. In the majority of cases, vasa praevia was detected during inspection and detection of placental abnormalities. The patients were either referrals with the suspicion of vasa praevia, or placental abnormalities and vaginal bleeding. After diagnosis, an individualized management for each patient with vasa praevia was formulated. For detailed patients’ characteristic and time of diagnosis, see Tables 1 and 2.

**Table 1:** Antenatally diagnosed pregnancies with vasa praevia and outcomes

Case	Diagnosis	Admission to hospital	Administration of corticosteroids	Mode of delivery	Gestational age at delivery	Birth weight (g)	APGAR	pH	Placental anomalies
1	29 + 2	32 + 3	–	Elective CS	34 + 5	2120	9/9/9	7.38	Total bipartite placenta praevia
2	36 + 1	36 + 4	–	Elective CS	36 + 6	2920	10/10/10	7.36	–
3	30 + 1	33 + 2	33 + 2/33 + 3	Elective CS	35 + 6	2730	7/8/10	7.29	Placenta praevia totalis
4	25 + 2	34 + 6	34 + 6/35 + 0	Elective CS	35 + 0	2790	9/10/10	7.23	Low-lying placenta
5	23 + 1	33 + 5	33 + 5/33 + 6	Elective CS	35 + 3	2490	7/8/9	7.34	Bipartite placenta
6	16 + 0	27 + 3	27 + 3/27 + 4	Acute CS	30 + 5	1233 1340	9/9/9 8/8/8	– 7.35	Total placenta praevia of fetus I
7	27 + 2	28 + 2	–	Acute CS	28 + 2	1340 1200	7/9/9 5/7/7	– –	Partial placenta praevia of fetus I
8	36 + 0	36 + 0	–	Elective CS	36 + 1	2880	9/10/10	7.24	Low-lying placenta
9	33 + 6	n.k.	–	Elective CS	34 + 3	2310	9/10/10	7.35	Bipartite placenta
10	30 + 2	37 + 5	–	Elective CS	37 + 6	2730	9/10/10	7.34	Bipartite placenta
11	22 + 3	37 + 1	–	Elective CS	37 + 2	2630	9/10/10	7.30	–
12	32 + 4	33 + 5	33 + 5/33 + 6	Elective CS	34 + 0	2220 1640	9/9/10 9/10/10	7.31 7.31	– –
13	24 + 1	36 + 6	36 + 6/37 + 0	Elective CS	37 + 2	2960	9/10/10	7.31	Partial placenta praevia
14	32 + 3	37 + 0	–	Elective CS	37 + 0	2250	9/10/10	7.33	–
15	27 + 4	36 + 6	–	Elective CS	36 + 6	2430	7/9/10	7.32	–
16	27 + 1	26 + 6	26 + 6/27 + 0	Elective CS	37 + 5	3230	7/7/9	7.12	Marginal placenta praevia
17	33 + 6	36 + 6	–	Elective CS	36 + 6	3090	8/8/9	7.25	Partial placenta praevia, Bipartite placenta
18	24 + 6	37 + 4	–	Elective CS	37 + 4	2800	9/10/10	7.31	Marginal placenta praevia
19	28 + 2	28 + 2	28 + 2/28 + 3	Elective CS	34 + 2	2400 2640	9/10/10 9/10/10	7.36 7.32	Vasa praevia fetus 1

*CS* caesarean section, *n.k.* not known, *IVF* in vitro fertilization, *administration of corticosteroids* Gestational age during application of steroids for lung maturation, *GA* gestational age

**Table 2:** Patients’ characteristics

Case	Age (y)	BMI	Nicotine	Conception	Parity	GDM	PE
1	36	24.3	No	Spontaneous	2	No	No
2	39	27.7	No	Spontaneous	3	No	No
3	34	24	No	Spontaneous	6	No	No
4	33	23	No	Spontaneous	1	No	No
5	34	30	No	IVF	1	No	No
6	35	16	Yes	IVF	1	No	No
7	36	28.7	No	IVF	1	No	No
8	31	20.1	No	Spontaneous	1	No	No
9	40	15.8	No	Spontaneous	1	No	No
10	38	21	No	Spontaneous	1	No	No
11	38	21	No	Spontaneous	3	No	no
12	36	20.5	No	IVF	1	No	No
13	33	33.7	No	IVF	1	No	No
14	30	22.1	No	Spontaneous	1	No	No
15	33	26.8	No	Spontaneous	1	No	No
16	35	22.9	No	Spontaneous	2	No	No
17	31	23.8	No	Spontaneous	1	No	No
18	31	19.7	No	Spontaneous	3	No	No
19	39	20.5	No	IVF	1	No	No

*y* years, *BMI* body mass index, *GDM* gestational diabetes mellitus, *PE* pre-eclampsia

After vasa praevia was detected, patients underwent close follow-up examinations, including weekly evaluations of possible risk factors which include uterine contractions, vaginal bleeding, cervical insufficiency, and placental abnormalities. In literature, over 80% of vasa praevia are associated with placenta praevia, conception by assisted reproductive technologies, a bilobed or succenturiate placenta, umbilical cord insertion in the lower third part of the uterus at first-trimester ultrasound and velamentous cord insertion [5]. Patients with a presumed low risk for preterm birth were delivered by caesarean section between 35 + 0 and 37 + 0 weeks of gestation. Thus, obligatory administration of corticosteroids for lung maturation was not necessary. Patients with a presumed high risk for preterm birth were delivered by caesarean section in an earlier week of gestation after administration of corticosteroids to promote foetal lung maturation. An individual decision on the ongoing management regimen was made by a team of obstetricians in an obstetric board/case conference according to placental abnormalities and supply of the fetus, foetal growth, possible bleeding episodes, cervical length, frequency of contractions and mental condition of the affected women with the aim to prolong the pregnancy.

## Results

Nineteen cases of vasa praevia were identified (15 singletons and four twin pregnancies). Details are provided in Table 1. All cases were diagnosed antenatally. In 13 patients (79%), placental anomalies were found (details Table 1). In all cases, the mode of delivery was a caesarean section. Apart from 17 planned procedures, there were just two emergency caesarean deliveries: one due to premature abruption of the placenta (case number 7). Another caesarean section was performed at week 30 + 5 (case number 6), earlier than planned, due to increased vaginal bleeding in a woman with placenta praevia totalis and vasa praevia of one fetus in a dichorionic twin pregnancy. There was no foetal death and none of the newborns needed blood transfusion in any case. Moreover, foetal outcome after acute caesarean section was adequate, with Apgar scores of 9/9/9 and 8/8/8 (case 6) vs. 7/9/9 and 5/7/7 (case 7). Unfortunately, according to our documentation system, pH values could not be evaluated due to clotting; therefore, we cannot provide any information.

The earliest caesarean delivery was performed at week 28 + 2 of gestation as an emergency caesarean section, because of preterm abruption of the placenta in a dichorionic twin pregnancy (case 7). The latest elective caesarean delivery took place at 37 + 6 weeks of gestation (case number 10). The application of corticosteroids for foetal lung maturation depended on gestational age at delivery and medical history (e.g. clinical signs of preterm birth). Therefore, just 8 of the 19 patients received corticosteroids. Those eight cases, receiving corticosteroids, had episode of vaginal bleeding or clinical signs for preterm birth, such as shortening of the cervix and/or uterine contraction.

## Discussion

In our study, all cases were identified antenatally through ultrasound examination. Despite two emergency caesarean sections, due to premature abruption of the placenta and increased vaginal bleeding with placenta praevia, there was no foetal death and no foetal blood transfusion in any of our individually managed cases.

The feasibility of prenatal screening for umbilical cord insertion, which enables the detection of vasa praevia during first trimester screening, has already been demonstrated [8, 9]. In the mid-trimester, detection is also possible with a high overall predictive reliability (sensitivity 100%, specificity 99.8%), as reported previously [10]. Recently, Hasegawa et al. suggested that sonographic screening in the late first or early second trimester, with follow-up examinations in cases with a low cord insertion in the second trimester, would be a useful way to detect vasa praevia [8]. Although the benefits of prenatal diagnosis have been suggested [8–10], there are no data from randomized trials that would provide highly reliable data upon which to base recommendations. Therefore, it is not surprising that there is a lack of consensus concerning the management of pregnancies with vasa praevia. Available guidelines differ from each other and do not provide a consistent method of managing these cases. Management strategies often depend on institutional policy. Universal screening is generally not recommended, although the feasibility of prenatal screening for vasa praevia and detecting this condition has already been demonstrated, as mentioned above. However, it has been recommended that the placental cord insertion in patients with risk factors for vasa praevia should be evaluated during the second-trimester scan [3, 4, 6, 7, 11, 12]. A few studies and guidelines consider the optimal time for caesarean delivery to be between 34–35 weeks after early hospitalization at about 28–32 weeks of gestation, and administration of corticosteroids to promote foetal lung maturation [3, 4, 6, 7, 13]. A study published by Golic et al. in 2013 recommended an individual, risk-adapted management of vasa praevia and an elective caesarean section between 35 and 37 weeks and no obligatory administration of corticosteroids by delaying the caesarean section up to two weeks beyond the standard recommendation [14]. This study group described a management strategy similar to ours and reported comparable results.

An individualized management which delays delivery for up to one to two weeks beyond 35 weeks of gestation, if possible, considerably results in a better outcome for the newborn, due to the fact that the newborn has more time for overall and lung maturation and potential long-term side effects of corticosteroids such as impaired neurological development and problematic behaviour decreases [14]. On the other hand, one might argue that such a management might increase the risk of premature rupture of the membranes and, thereby, the risk of foetal death due to exsanguination. Robinson et al. found no benefit to expectant management beyond 37 weeks of gestation [13] However, in our case series, two emergency caesarean sections were performed because of premature abruption of the placenta (and not of the membranes) and vaginal bleeding because of placenta praevia totalis.

Nevertheless, there is limited evidence available to determine the ideal timing of delivery. On the basis of our data, our suggestion for the diagnosis and the management of vasa praevia would be (summary Table 3):

- documentation of the insertion of the umbilical cord in the course of the 1st trimester screening;
- confirmation of the insertion of the umbilical cord during second-trimester screening including screening for vasa praevia with transvaginal ultrasound and Doppler, as well as other placental abnormalities and low-lying placentas;
- if vasa praevia is present, a risk evaluation (e.g. risk factors for preterm birth, placental abnormalities, uterine contraction, vaginal bleeding) should be performed followed by weekly follow-up examinations with re-evaluation of possible risk factors;
- Patients at high risk for preterm birth, caesarean section should be performed between 34–35 weeks after early hospitalization at about 30–32 weeks of gestation and administration of corticosteroids to promote foetal lung maturation, whereas in patients at low risk for preterm birth, caesarean section can be delayed to 35–37 weeks after hospitalization at about 32–34 weeks of gestation. Thus, administration of corticosteroids would not be obligatory in the latter cases. Weekly follow-up examinations until caesarean section should be maintained.

**Table 3:** Diagnosis and management of vasa praevia

1st trimester screening	Placenta position Umbilical cord insertion
2nd Trimester	Foetal anomaly scan Screening for placenta abnormalities and low lying placenta Confirmation of umbilical cord insertion Screening for vasa previa
Vasa previa	With risk factors*: 1. weekly controls, 2. hospital admission at 30–32 weeks, 3. administration of corticosteroids, 3. caesaren section between 34–35 weeks of gestation Without risk factors: 1. weekly controls, 2. hospital admisson at 32–34 weeks, 3. caesaren section between 35–37 weeks of gestation
Placenta	Normal placenta: routine care Placental abnormalities and low lying placenta: re-scan at 32 weeks of gestation

Suggestions for diagnosis and individual risk-adapted management of vasa praevia, which attempts to delay the timing of caesarean section up to 2 weeks beyond the standard recommendation

Risk factors*: signs for preterm birth (i.e. vaginal bleeding, uterine contractions, shortening of the cervix) and placenta abnormalities, signs of placental dysfunction and history of placental dysfunction and multiple pregnancy

The obvious limitation to our report is the small sample size. Due to the fact that vasa praevia is still a rare event and not every woman receives a placental examination in our department. Moreover, we are aware of the fact that an individualized treatment regimen was chosen for each affected woman. Thus, we cannot list in detail cutoff values for the different follow-up examinations that made the obstetric board decide on whether caesarean section was delayed.

## Conclusion

However, our data suggest that an individualized treatment with delayed caesarean section is feasible. In fact, our study and other reports on this topic make clear that protocols for diagnosis and management of vasa praevia significantly improve the outcome for both mother and child [15]. The fact that management modalities of vasa praevia are non-uniform in literature indicates an important need for further research to establish a standardized, consistent management for cases of vasa praevia. Due to the fact that vasa praevia can put the pregnant woman and her fetus in high-risk situations and that vasa praevia is detectable during screening for abnormal cord insertion and low lying placenta, we consider such a screening program useful.
